# Effect of different reheating processes and conditions on the nutritional, functional, and microbiological properties of cow meat

**DOI:** 10.1002/fsn3.4083

**Published:** 2024-03-18

**Authors:** Ombotoh Sabastin Nanje, Veshe‐Teh Zemoh Sylvia Ninying, Fabrice Tonfack Djikeng, Theresia Azia Morfor, Aduni Ufuan Achidi

**Affiliations:** ^1^ School of Agriculture and Natural Resources Catholic University Institute of Buea Buea Cameroon; ^2^ Department of Public Health and Administration, Nutrition and Dietetics, School of Health Science Biaka University Institute of Buea Buea Cameroon; ^3^ Department of Biochemistry and Molecular Biology, Faculty of Science University of Buea Buea Cameroon

**Keywords:** cow meat, functional property, microbial count, nutritional composition, reheating

## Abstract

The objective of this study was to evaluate the effect of different reheating methods and conditions on the proximate composition, mineral content, oil quality, and functional and microbiological properties of cow meat. For this, a survey was carried out to identify the reheating methods used in the community. For this study, 8.6 kg of raw beef was used and group 1 (500 g) served as raw control. The remaining 8.1 kg was boiled for 30 min in 5 L of water. Four hundred grams of boiled beef was removed and served as cooked control (group 2). The remaining 3688 g was divided into four groups of 922 g, which were, respectively, divided into subgroups of 307 g. The four sets of subgroups were, respectively, reheated by boiling, frying, microwaving and oven roasting for 3 days. Reheating was done three times a day and samples were collected at the end of each day for further analysis. Changes in proximate composition, mineral content, oil quality, functional properties, and microbiological count were evaluated using standard methods. Results showed that frequent reheating of food was the most used preservation method of cooked food, and boiling and steaming were the most used methods. Reheating time significantly reduces the protein, mineral content, and oil quality of cow meat in general. For the functional properties, reheating methods/conditions generally increased the water‐holding capacity, loose and packed bulk densities as well the pH of the meat. Reheating by frying increased the porosity and Hausner ratio of the meat powder while all the reheating treatments reduced the swelling capacity and titratable acidity of cow meat powder. Generally, the reheating methods and duration significantly reduced the bacterial count of cow meat powder. Cow meat should not be reheated for more than 2 days in order to preserve its physiochemical properties.

## Graphical abstract

Over‐reheating of meat significantly affects its proximate composition, mineral content, and oil quality. Over‐reheating of meat considerably impacts the functional properties of its proteins. Reheating reduces the bacterial load of cow meat.

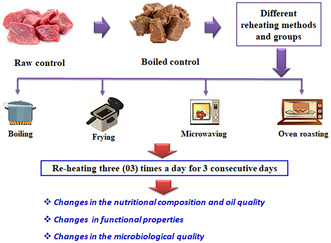

## 1 INTRODUCTION

Meat is a class of food that represents a very important part of the human diet. It is flesh obtained from animals such as cows, pigs, goats, and poultry. It has very strong economic, cultural, and health implications (Biesalski, 2005). It is an important source of nutrients, including proteins, carbohydrates, lipids, vitamins, and mineral elements (Pereira & Vicente, 2013). Worldwide, meat and its products are considered important sources of high biological proteins, vitamins, and minerals in the human diet. The nutritional content of meat depends on the animal origin and the part of cut (Font‐I‐Furnols & Guerrero, 2014; Van Loo et al., 2010). Consumption of meat varies among and within countries based on intrinsic and extrinsic factors influencing consumers' preferences and perceptions. Meat variety is often chosen with respect to quality, nutritional composition, and constantly evolving feeding habits. This has constantly put a distinctive amount of pressure on the beef market due to its high solicitation in various parts of the world (Henchion et al., 2014). Previous studies have illustrated the nutritional composition of meat to be 76% moisture, 20.80% protein, 2.54% fat, 0.61% ash, and 0.35% carbohydrate; with that of fresh lean beef are 75% moisture, 22.3% protein, 1.8% fat, and 1.2% ash (Bakht et al., 2004). Cameroon occupies a prominent place in both sub‐Saharan Africa and the subregion of the Central African area, due to its large livestock production rates with approximately 5.001 million cattle and meat consumption of 13.3 kg/per capita/year (FAOSTAT, 2014). In Cameroon, beef consumption is most prominent in the North, especially in Maroua with daily consumption of beef estimated at 133 g/capita, which is 10 times higher than the worldwide average meat consumption (Ritchie et al., 2017; Vougat et al., 2016). Aside from meat being a good source of both macro‐ and micronutrients, it contains some healthy compounds such as creatine, taurine, glutathione, and conjugated linoleic acid which have positive impact on human health (Candow et al., 2014; Spriet & Whitfield, 2015). Due to its rich nutrient content, meat is very perishable as its susceptibility to microorganism proliferation is extremely high (Zerabruk et al., 2019). Typical spoilage microorganisms here include *Escherichia coli, Salmonella* spp., *Lactobacillus* spp., *Clostridium* spp., *Leuconostoc* spp., etc. generally responsible for off odors, discoloration, sliming, and food‐borne diseases (Nychas et al., 2008). As such, there is development of new strategies to process and preserve meat with regards to quality and shelf life, with the most common methods being boiling, frying, oven roasting, and microwaving (Jorge et al., 2003).

In Cameroon, many households lack the means to preserve and store raw and cooked food items. In some areas where these facilities are available, constant electric breakdown can lead to significant losses of easily perishable products such as fish, meat, and vegetables (MINEPIA, 2021; OIE, 2015). In the majority of households and university student residential areas, foods are generally reheated for preservation. In some cases, cooked food can be consumed for about 3–4 days with constant reheating as preservation technique. The methods locally used include boiling, steaming, oven heating, microwaving, and pan frying (McAfee et al., 2010). Constant reheating of food, although it inhibits microbial growth, can significantly alter the nutritional properties of the food sample (Tornberg, 2005). Constant heating promotes the development of nonenzymatic browning reactions that significantly reduce the carbohydrate and protein/amino acid content of foodstuffs (Djikeng, Selle, et al., 2018; Djikeng, Teyomnou, et al., 2018). In the same line, lipid oxidation reaction will be activated and will lead to a significant decrease in essential fatty acids and fat‐soluble vitamins, as well as to the formation of toxic substances that can cause disorders such as cardiovascular diseases, mutagenesis, and cancers and destroy food flavor (Cuvelier & Maillard, 2012; Domínguez et al., 2015). It is therefore important to know to what extent foodstuffs, especially beef which is used in many dishes in Cameroon, can see their nutritional, sensory, and microbiological properties being affected by frequent reheating.

In past studies, the impact of reheating on food quality characteristics was reported. Meng et al. (2022) examined the factors affecting the quality of beef meatballs during processing and preservation. Seerley (1966) evaluated the effect of reheating on the nutritional composition of grains. Along the same line, the influence of reheating methods on the physicochemical characteristics and warm‐over flavor of nutmeg extract‐enriched precooked beef meatballs was investigated by Parvin et al. (2020). Xie et al. (2022) evaluated the effect of heating methods on the nutritional quality and digestive characteristics of Chinese steamed bread. The impact of reheating on the tenderness of beef *longissimus lumborum* and *biceps femoris* was evaluated by Obuz et al. (2003). Although studies have been carried out on the impact of reheating on the sensory and nutritional characteristics of some food, there is relatively no information on the impact of reheating using the local methods applied in Cameroonian households on the nutritional, functional, and microbiological properties of beef.

The objective of this study was to determine the effect of different reheating methods and conditions on the proximate composition, mineral content, oil quality, and functional and microbiological properties of cow meat.

## 2 MATERIALS AND METHODS

### 2.1 Materials

Raw fresh beef (10 kg) was purchased from a slaughterhouse located in Muea, South‐West Region, Cameroon. All reagents and chemicals used were of analytical grade.

### 2.2 Study area

The study was conducted in Buea, the southwest region of Cameroon. Buea is a city of the Fako division situated at an elevation of 3000 feet (900 m) above sea level. Its coordinates are latitude 4.159302, longitude 9.243536, DMS Lat 4°9′33.4872″ N, and DMS Long 9°14′36.7296″ E. It occupies a total land surface of 870 km^2^. It is located on the southeast slope of Mount Cameroon.

### 2.3 Methods

#### 2.3.1 Survey on the different reheating methods locally applied in the Buea municipality

In order to identify the commonly used reheating methods applied in households and university residential areas; a survey was carried out on 320 women aged 21 to 50 years. The consent of each participant was obtained before administering the questions and their information was kept confidential.

#### 2.3.2 Study design and sample collection

Raw beef (fresh) was purchased from the slaughterhouse and put in boxes containing ice. The boxes were transferred to the laboratory where the beef was divided into different groups which were boiled. The boiled samples were reheated using different methods (boiling, frying, oven, and microwave reheating). The processed beef samples including the controls were dried and ground for the analysis of their nutritional, functional, and microbiological properties.

#### 2.3.3 Effect of different reheating conditions on the nutritional value of cow meat

##### Sample preparation

Raw beef sample (8.6 kg) was used in this study. Five hundred grams was used as raw control (group 1) and the remaining 8.1 kg was boiled in 5 L of water at about 98°C for 30 min. After cooling, the beef was cut into small pieces and the boiled beef mass was 4088 g. About 400 g of boiled beef was used as cooked control (BM). The remaining 3688 g of boiled beef was divided into four groups of 922 g each, namely groups 3, 4, 5, and 6. Group 3 consisted of three subgroups of 307 g each, which were, respectively, reheated by immersion boiling (MRB) three times a day (morning, afternoon, and evening) for 3 days. Group 4 consisted of three subgroups of 307 g each, which were, respectively, reheated in a microwave (MRMW) three times a day as previously mentioned for 3 days. Group 5 consisted of three subgroups of 307 g each, which were, respectively, reheated in the oven (MROR) three times a day as previously stated and for 3 days. It is important to note that, samples were collected at end of each day, and dried in the oven at 50°C till constant weight before being ground and the powders used for further analysis. Reheating was performed for 10 min at each session.

##### Effect of reheating on the nutritional value of cow meat

###### Proximate composition

The analysis of proximate composition was carried out using the Association of Official Analytical Chemists methods (AOAC, 1990). The parameters determined were the ash, fiber, protein, and fat contents only. The samples were incinerated at 550°C following the AOAC procedure 942.05. The micro‐Kjeldahl method was used to determine the nitrogen content following the AOAC procedure 984.13 and the protein was estimated as nitrogen × 6.25. The Soxhlet method was used for the measurement of the fat content according to the AOAC procedure 963.15. The AOAC method (AOAC, 2005) was used for the determination of the fiber content.

###### Mineral content

The ash obtained from the incineration of each sample was dissolved with 10 mL of a 20% HCl solution. The solution was filtered and the filtrate was used for the detection and quantification of minerals. An atomic absorption spectrometer (Varian 220FS Spectra AA, Les Ulis, France) was used for this purpose and for the following minerals: calcium, sodium, iron, potassium, and magnesium. The vanadomolybdate colorimetric method was used for the determination of the phosphorus content. Calibration curves of standards were used for this purpose.

##### Effect of reheating on cow meat oil quality

###### Oil extraction

Oils from processed and unprocessed meat samples were extracted using the maceration method as reported by Womeni et al. (2013). About 100 g of meat was macerated in 400 mL of hexane. The mixture was regularly stirred for 48 h before being filtrated with the Whatman paper (Number 1). The residues were further macerated in 200 mL of hexane to extract the residual oil. The filtrate was evaporated at 40°C under vacuum on a rotatory evaporator for the removal of hexane. The oil was weighed for the determination of the extraction yield.

###### Oil characterization

The extracted oil samples were characterized by determining their peroxide, *p*‐anisidine, and TOTOX values. The peroxide value was determined following the IDF standard method 74A: 1991 (International IDF Standards, 1991). The *p*‐anisidine was determined using the AOCS method CD 18–90 (AOCS, 2003). The TOTOX value was calculated from the *p*‐anisidine and peroxide values following the equation: TOTOX = 2PV + AV (Shahidi & Wanasundara, 2008).

##### Effect of reheating on some functional properties of cow meat proteins

###### Water‐ and oil‐holding capacity

The methods described by Lin et al. (1974), modified by Tambo et al. (2019), were used for the determination of these parameters. About 1 g of meat powder was mixed with 10 mL of palm olein for the evaluation of the oil‐holding capacity. The same method was used for the water‐holding capacity but in this case distilled water was used. The mixture was incubated in a water bath at 30°C for 30 min before being centrifuged at 4500 *g* for 15 min. The volume of water or oil absorbed was measured. The WHC and OHC were determined as follows:

$$\mathrm{WHC}/\mathrm{OHC}=\frac{\mathrm{Vi}-\mathrm{Vf}}{\mathrm{Vi}}\times 100$$

where vi = Initial volume of water/oil and vf = volume of water/oil after centrifugation.

###### Loose and packed bulk density

The method reported by Okaka et al. (1992) was used for the determination of these parameters. About 20 g of meat powder was introduced into a 100 mL measuring cylinder and the volume occupied by the sample recorded. After that, the cylinder was tapped 100 times and the second volume recorded. These values were used to calculate the loose and packed bulk densities using the formula:

$$\begin{array}{c} \text{Loose density},\text{Packed density}=\text{Weight of Sample}\\ \;/\text{Volume occupied}\;\mathrm{by}\;\text{the sample} \end{array}$$

###### Hausner ration and porosity

The Hausner ratio which is the proportion of loose and packed bulk densities was calculated as follows:

Hausner ratio=Packed density/Loose density

The following formula was used for the calculation of the porosity, equally from the loose and packed bulk densities:

$$\begin{array}{c} \text{Compressibility index}/\text{Porosity}\\ \;=\left(\text{Packed density}-\text{Loose density}/\text{Packed density}\right)\times 100 \end{array}$$

###### Swelling capacity (SC)

Meat powder solutions (10%) (w/v) were prepared and incubated in the water bath for 30 min at 30°C. The mixture was centrifuged at 4500 *g* for 15 min. The swelling capacity (SC) was estimated as the difference between the weight of the sample that has retained the water (*W*_1_) and that of the initial sample (*W*_0_). The SC was calculated using the following equation. The swelling capacity of meat was determined according to the method described by Okezie and Bello (1988), modified by Tambo et al. (2019).

$$\mathrm{SC}=\frac{\left[\left(W_{1}-W_{0}\right)\times 100\right]}{W_{1}}$$

###### pH

The AOAC (1990) method was used for the measurement of the pH of meat powder solutions. About 1 g of each sample was introduced into centrifuge tubes and mixed with 10 mL distilled water. The mixture was stirred for 30 min using a vortex and centrifuged at 4500 *g* for 15 min. The pH of the aqueous phase was determined using a calibrated pH meter at room temperature (25°C).

###### Titratable acidity

The method reported by AFNOR (French Association for Standardization) (1981) was used for the determination of the titratable acidity. About 1 g of sample was soaked in 10 mL of distilled water for 30 min and the volume of solution taken to 50 mL. After that, 0.1 mL of phenolphthalein solution (0.05% in ethanol 1/1 (v/v)) was added. The mixture was titrated using a 0.1 N NaOH solution (4 g/L). When a persistent pink color (for at least 30 s) appeared, titration was stopped and the volume of NaOH used was recorded (V_NaOH_ in mL). The titratable acidity was calculated as follow:

$$\%\text{Titratable acidity}=\frac{\mathrm{mL}\times N\times 90\times 100}{m\times 1000}$$

where mL = mL 0.1 NaOH used; *N* = normality of 0.1 N NaOH; and *m* = mass of flour.

##### Bacterial count

###### Sample preparation

Samples were processed according to the ISO 6579‐1 method (ISO, 2013). In this procedure, 6.6 g of plate count agar (PCA) was weight and introduced into two conical flasks of 500 mL each, followed by the addition of 300 mL of distilled water. The flasks were sealed with aluminum foil paper, and the mixture was homogenized vigorously for about 20 s and allowed to stand inside an autoclave. Nine milliliter of distilled water was introduced into 42 test tubes and sealed using cotton and introduced into an autoclave containing two conical flasks of diluted plate count agar. The medium was boiled for a few seconds until the ingredients were completely dissolved and then sterilized in an autoclave at 121°C for 15 min. After autoclaving, the PCA medium was allowed to cool down to a temperature range of 45–50°C.

###### Sample dilution

Three series of decimal dilutions were prepared (10^−3^, 10^−4^, and 10^−5^) for the samples to be analyzed. Starting with the original sample, 1 g of sample was transferred into the first dilution tube containing 9 mL of distilled water and the test tube was covered using aluminum foil paper. The mixture was stirred to dissolved, and then 0.1 mL was transferred from the first dilution tube to the second. This was done for all the 14 samples.

###### Inoculation

Using a 0.1 mL micropipette, 0.1 mL of sample was transferred from each dilution into the surface of all the 42 petri dishes. Once the plate count agar medium had reached the desired temperature, it was mixed to ensure homogeneity. Then, the medium was poured into sterile petri plates and moved back and forth across the surface of the dish for an even distribution of inoculum.

###### Incubation

After inoculating the samples were put in a heating incubator of type DHP‐9052 at a temperature of 37°C for 24 h. After incubation, the plates were examined, and bacterial colonies were counted. The results were reported as colony‐forming units (CFU) per milliliter or gram, taking into account the dilution factors used during the procedure. The total mesophilic aerobic count was determined using the pour plate method (ISO, 2023).

###### Plate reading

The colony‐forming units (CFU) appearing on the petri dishes after the incubation period were counted. Only plates with colony‐forming units between 30 and 300 were considered. All experiments were performed with three replications and the results were expressed as colony‐forming units per gram of fresh beef (CFU/g).

#### 2.3.4 Statistical analysis

The obtained data were subjected to one way analysis of variance (ANOVA) with the Student–Newman–Keuls tests using the software Graphpad‐InStat version 3.05 to evaluate the statistical implication of the data. The variances were important at probability level less than 5%.

## 3 RESULTS AND DISCUSSION

### 3.1 Survey

**FIGURE 1:**
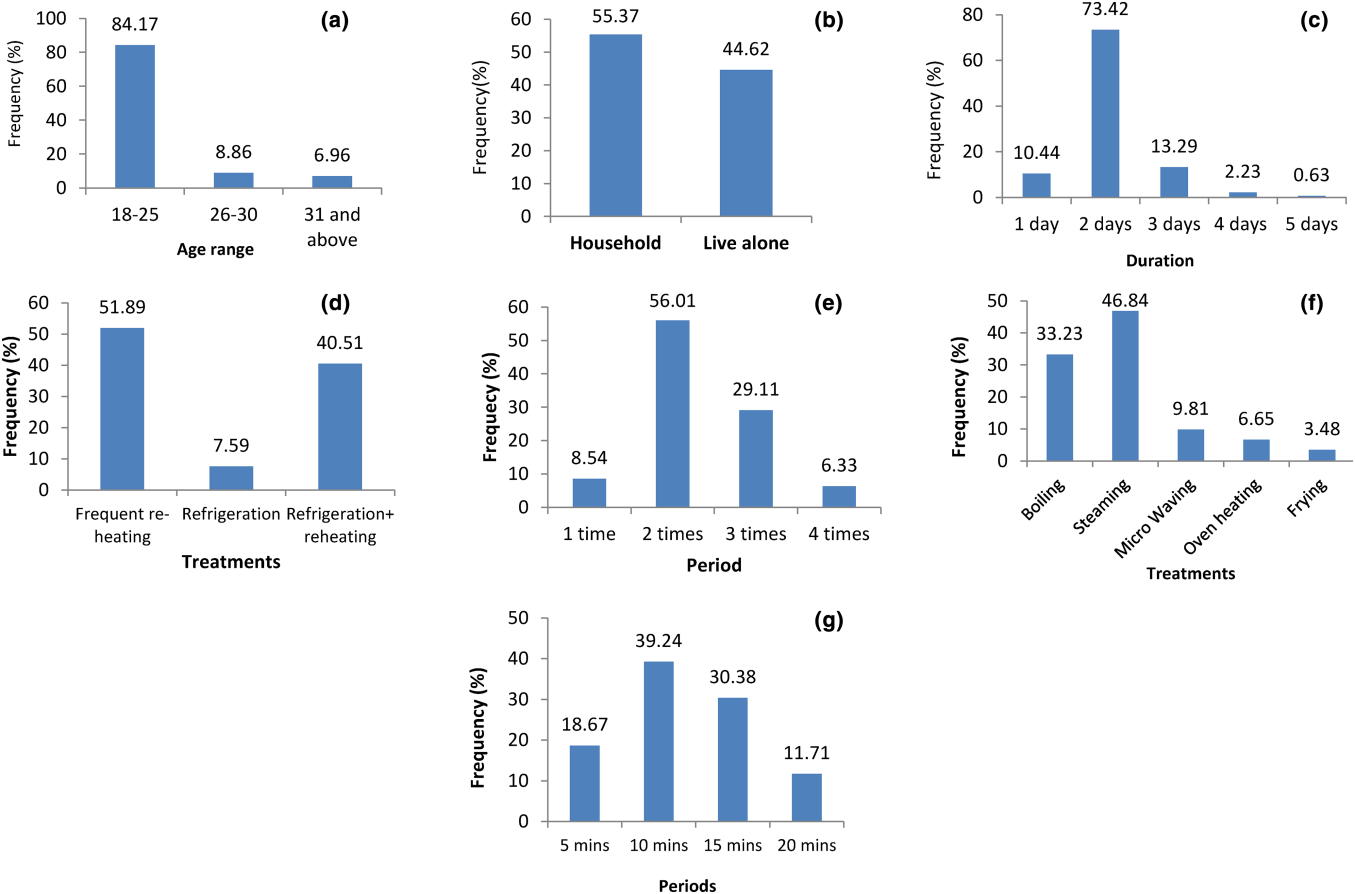
(a‐g) Age distribution of participants (a); residential conditions of participants (b); time taken to consume prepared food with meat by participants (c); preservation methods of food prepared with meat by participants (d); daily frequency of reheating prepared food with meat for preservation purpose (e); reheating methods on food prepared with meat (f); and reheating time frequency of food prepared with meat (g).

In many African households, especially in rural areas, the facilities for food preservation are absent, but local methods such as reheating prepared food are applied to extend their shelf life. In urban areas, a wide range of preservation methods are used due to the availability of facilities, but sometimes, the population is forced to use reheating to preserve food due to unstable electricity. The data obtained from the survey are presented in Figure 1a‐g. It can be observed that 84.17% of participants aged 18–25 and only 8.86% and 6.69% aged 26–30 and 31 above, respectively. 55.37% of participants were found to live in households and 44.62% alone. 73.42% of the participants were found to take 2 days to consume food with meat, while 13.29% took 3 days, 10.44% 1 day, 2.23% 4 days, and 0.63% 5 days. The lack of financial resources and time to prepare food every day can justify the preparation of enough food followed by its consumption in 2 days or more. 51.89% were found to frequently preserve their food by reheating, 40.51% by refrigeration plus reheating, and 7.59% by refrigeration only. This can be explained by the lack of facilities that can be used to preserve food in some households and student rooms due to financial constraints. It was also observed that 56.01% of participants reheat their food twice per day, 29.11% thrice, 8.54% once, and 6.33% quadruple per day. These reheating frequencies can be justified by the prevention of microbial spoilage. 46.84% of surveyed individuals reheat their food by steaming, 33.23% by boiling, 9.81% by microwaving, 6.65% by oven heating, and 3.48% by frying. The high frequency of boiling and steaming can be explained by their accessibility to people since they are cheap and simple reheating methods. 39.24% reheat their food prepared with meat for 10 min, 30.38% for 15 min, 18.67% for 5 min, and 11.71% for 20 min. These ranges are considered by the population as reasonable to inhibit spoilage.

### 3.2 Proximate composition

The effect of different reheating methods on the ash, fiber, protein, and lipid contents of cow meat is presented in Table 1. Ash, lipid, protein, and fiber contents were found to be ranged between 0.96% and 1%; 8.12% and 24.79%; 30.06% and 61.69%; and 2.32% and 9.30%. No significant (*p* > .05) difference was recorded in the ash content of all samples. The ash content obtained in this study was similar to 1.10% reported by Shehzad et al. (2014), while the protein and fat contents were higher than 25.84% and 2.05%, respectively, obtained by these same authors. Ash content generally informs on the amount of minerals present in the food sample. Samples reheated by frying presented higher (*p* < .05) lipid content compared to both raw (RM) and boiled (BM) controls and other processed samples. This can be attributed to the oil absorbed during the frying process. Similar results were obtained by Djikeng et al. (2022) with snail meat powder. On the other hand, samples reheated by frying and oven roasting significantly (*p* < .05) increased the protein content of cow meat compared to the boiled (BM) and raw (RW) controls and other reheating techniques. However, it significantly (*p* < .05) decreased with the extension of reheating time with boiling (MRB), oven roasting (MOR), and microwaving (MRWMW). This can be attributed to the involvement of proteins in nonenzymatic browning reactions. The significant (*p* < .05) increase in protein content was recorded with fried samples and other reheated samples, especially on the first day of reheating can be explained by the release of bound proteins due to heat. Similar results were recorded by Tenyang et al. (2022) who reported that boiling, frying, smoking, and steaming significantly (*p* < .05) increase the protein content of *Polypterus bichir bichir* fish. A significant (*p* < .05) decrease in fiber content was registered in all processed samples compared to the raw control (RM). This can be explained by the degradation of fibers into smaller compounds due to heat (Căpriţă et al., 2011).

**TABLE 1:** Effect of different reheating conditions on the proximate composition of cow meat (%).

Samples	Ash	Lipid	Protein	Fiber
RM (Control)	1.00 ± 0.00^a^	15.97 ± 1.12^a^	35.88 ± 1.01^a^	9.30 ± 0.31^a^
BM (Control)	0.97 ± 0.03^a^	13.57 ± 0.21^ab^	45.34 ± 0.22^b^	3.34 ± 0.10^b^
MRB1	0.98 ± 0.01^a^	11.48 ± 0.33^bc^	46.38 ± 0.87^b^	4.85 ± 0.21^b^
MRB2	0.97 ± 0.02^a^	8.12 ± 0.12^c^	30.94 ± 0.78^c^	3.41 ± 0.11^bc^
MRB3	0.96 ± 0.01^a^	16.54 ± 0.13^a^	30.63 ± 1.01^c^	3.15 ± 0.30^bc^
MRF1	0.99 ± 0.00^a^	24.79 ± 2.04^de^	30.06 ± 0.91^c^	3.27 ± 0.11^bc^
MRF2	0.98 ± 0.01^a^	24.25 ± 0.23^d^	53.63 ± 1.04^d^	3.34 ± 0.22^bc^
MRF3	0.96 ± 0.03^a^	20.54 ± 0.15^e^	59.50 ± 0.43^e^	3.58 ± 0.32^bc^
MROR1	0.97 ± 0.01^a^	17.22 ± 0.22^a^	60.73 ± 0.73^e^	3.87 ± 0.11^bc^
MROR2	0.98 ± 0.00^a^	14.64 ± 0.71^a^	61.69 ± 2.04^e^	3.05 ± 0.09^bc^
MROR3	0.99 ± 0.02^a^	15.88 ± 0.31^a^	53.81 ± 2.02^d^	4.51 ± 0.12^b^
MRMW1	0.99 ± 0.05^a^	13.50 ± 0.52^ab^	45.06 ± 1.13^b^	2.82 ± 0.11^c^
MRMW2	0.96 ± 0.06^a^	15.07 ± 132^a^	30.94 ± 1.41^c^	2.58 ± 0.08^c^
MRMW3	0.97 ± 0.03^a^	16.10 ± 0.72^a^	31.75 ± 2.01^c^	2.32 ± 0.07^c^

*Note*: Data are presented as mean ± SD (*n* = 2) (a‐e) Values within the same column with different superscripts are significantly (*p* < .05) different.

Abbreviations: BM, boiled control (boiled meat); MFR1, meat reheated by frying on day 1; MFR2, meat reheated by frying on day 2; MFR3, meat reheated by frying on day 3; MRB1, meat reheated by boiling on day 1; MRB2, meat reheated by boiling on day 2; MRB3, meat reheated by boiling on day 3; MRMW1, meat reheated by microwaving on day 1; MRMW2, meat reheated by microwaving on day 2; MRMW3, meat reheated by microwaving on day 3; MROR1, meat reheated by oven roasting on day 1; MROR2, meat reheated by oven roasting on day 2; MROR3, meat reheated by oven roasting on day 3; RM, boiled control (raw meat).

### 3.3 Mineral content

The effect of different reheating methods on the mineral content of cow meat is exhibited in Table 2. Results showed Ca, P, Mg, Fe, Na, and K contents ranged between 240 and 480; 150 and 315; 4.86 and 87.48; 0.49 and 5.99; 10.20 and 58.80; and 217.10 and 598.00 mg/100 g, respectively. The Ca, Fe, K, Mg, and P contents obtained in this study were significantly (*p* < .05) lower compared to the values reported by Oz & Kotan (2016) during the evaluation of the effect of different cooking methods on some quality criteria and mineral composition of beef steak. Generally, for each of these minerals and each reheating method, a significant (*p* < .05) decrease in their concentration was observed with reheating time. Ca, P, Na, and K presented significantly (*p* < .05) lower values after processing compared to the boiled (BM) sample and the raw controls (RM). This can be attributed to the leaching of these minerals out of the meat during processing. Concerning the Mg content, reheating by microwaving and reheating by oven roasting and boiling on day 3 significantly (*p* < .05) reduced the concentration in this parameter compared to the control. The loss of this mineral from the meat through the water during processing can explain these changes. However, its increase in some samples can be attributed to the release of bound magnesium by heat. A similar explanation can stand for the iron content of the boiled control (BM) compared to other processed samples and the raw control (RM). Even though most of these minerals decreased with processing, the amount was still significant except for the iron. These minerals are of great importance for the good functioning of the body. Calcium plays the role of messenger in signal transduction and it is involved in the blood‐clotting process (Mehas & Rodgers, 1997). Magnesium is one of the most important cofactors used in metabolic reactions and it is involved in the biosynthesis of protein, energy production, and trapping of calcium in the teeth (Mehas & Rodgers, 1997). Sodium and potassium help in the regulation of blood pressure and the good functioning of muscles and nerves. Potassium was also reported to have a direct relationship with hypertension (Etiosa et al., 2018). Iron is part of the respiratory pigment and its deficiency can lead to severe anemia (Loumouamou et al., 2010).

**TABLE 2:** Effect of different reheating conditions on the mineral content of cow meat (mg/100 g).

Samples	Ca	P	Mg	Fe	Na	K
RM (Control)	480.00 ± 2.06^a^	330.95 ± 1.02^a^	15.66 ± 0.12^a^	1.50 ± 0.01^a^	58.80 ± 1.21^h^	598.00 ± 4.23^a^
BM (Control)	460.00 ± 4.33^b^	315.92 ± 2.31^b^	58.98 ± 0.33^b^	5.99 ± 0.02^b^	49.80 ± 0.84^g^	506.90 ± 1.04^b^
MRB1	440.00 ± 1.02^c^	309.35 ± 1.25^c^	53.46 ± 0.21^c^	2.21 ± 0.12^c^	45.30 ± 1.23^f^	481.40 ± 0.88^c^
MRB2	400.00 ± 0.91^d^	305.94 ± 0.21^a^	16.92 ± 1.03^a^	2.07 ± 0.01^ac^	40.50 ± 1.98^f^	479.90 ± 3.12^c^
MRB3	401.00 ± 2.11^d^	150.77 ± 0.43^d^	4.86 ± 0.10^d^	0.58 ± 0.04^d^	13.80 ± 1.23^a^	296.90 ± 1.56^d^
MRF1	456.00 ± 1.77^b^	221.62 ± 2.05^e^	87.48 ± 0.28^e^	1.13 ± 0.02^a^	10.60 ± 0.94^a^	457.20 ± 2.04^e^
MRF2	407.00 ± 5.01^d^	155.26 ± 0.32^f^	82.62 ± 2.31^f^	0.62 ± 0.00^d^	29.30 ± 2.03^d^	337.00 ± 2.60^f^
MRF3	402.00 ± 3.02^d^	144.29 ± 2.10^g^	19.44 ± 0.85^g^	0.53 ± 0.20^d^	35.80 ± 1.20^e^	237.00 ± 1.37^g^
MROR1	409.00 ± 4.21^d^	244.57 ± 4.66^h^	34.02 ± 1.66^h^	1.45 ± 0.11^a^	18.80 ± 2.03^bc^	276.90 ± 0.78^h^
MROR2	320.00 ± 2.43^e^	175.71 ± 1.11^i^	14.58 ± 2.74^a^	1.21 ± 0.08^a^	18.80 ± 0.94^bc^	237.00 ± 2.13^g^
MROR3	240.00 ± 1.40^f^	145.28 ± 0.85^g^	4.86 ± 0.24^d^	0.74 ± 0.03^e^	18.40 ± 1.74^bc^	217.10 ± 5.55^i^
MRMW1	430.00 ± 2.02^g^	247.07 ± 2.78^h^	10.69 ± 0.69^i^	1.89 ± 0.15^a^	23.50 ± 2.13^cd^	417.00 ± 4.17^j^
MRMW2	320.00 ± 1.54^e^	209.15 ± 3.32^j^	9.72 ± 0.67^i^	1.14 ± 0.11^a^	18.40 ± 0.32^bc^	356.90 ± 2.45^k^
MRMW3	240.00 ± 3.65^f^	144.78 ± 2.41^g^	4.86 ± 0.42^d^	0.49 ± 0.03^d^	10.20 ± 2.21^a^	217.10 ± 0.74^i^

*Note*: Data are presented as mean ± SD (*n* = 2) (a‐k) Values within the same column with different superscripts are significantly (*p* < .05) different.

Abbreviations: BM, boiled control (boiled meat); MFR1, meat reheated by frying on day 1; MFR2, meat reheated by frying on day 2; MFR3, meat reheated by frying on day 3; MRB1, meat reheated by boiling on day 1; MRB2, meat reheated by boiling on day 2; MRB3, meat reheated by boiling on day 3; MRMW1, meat reheated by microwaving on day 1; MRMW2, meat reheated by microwaving on day 2; MRMW3, meat reheated by microwaving on day 3; MROR1, meat reheated by oven roasting on day 1; MROR2, meat reheated by oven roasting on day 2; MROR3, meat reheated by oven roasting on day 3; RM, boiled control (raw meat).

### 3.4 Oil quality

The analysis of oils and fats generally informs on their quality, especially their primary and secondary oxidation, as well as their hydrolytic activity. The changes in cow meat oil quality during reheating are presented in Table 3. Results showed peroxide, *p*‐anisidine, and TOTOX values ranged between 0.36 and 13.35 meq O_2_/kg, 3.00 and 24.02, and 3.72–46.95, respectively. The peroxide values obtained in this study were lower than 15 meq O_2_/kg, which is the recommended peroxide value for edible crude oils (FAO/WHO, 2009). A significant (*p* < .05) increase in peroxide value of cow meat oil was recorded with the treated samples compared to the raw control (RM). This can be attributed to the formation of hydroperoxides, which are the primary oxidation products released in oil when they are subjected to heat. In the same line, the *p*‐anisidine of oil obtained from processed samples was found to be significantly (*p* < .05) higher compared to the controls. The TOTOX value also significantly (*p* < .05) increased with processing and was more pronounced in the same samples that presented the highest anisidine values. Meat reheated by boiling, roasting, and microwaving exhibited significantly (*p* < .05) higher anisidine values compared to those reheated by frying. This parameter informs on the secondary oxidation state of oils marked by the formation of 2‐alkenal and 2, 4‐dienals. The fact that the samples reheated by frying had the lowest anisidine value compared to the other reheating methods shows that less secondary oxidation products were formed under this condition compared to other reheating methods. These samples were the least oxidized looking at their TOTOX values. Reheating by frying therefore preserved better the lipid quality of cow meat. These results are in agreement with the findings of Tenyang et al. (2022), who reported that boiling, frying, smoking, and steaming significantly (*p* < .05) increase the formation of primary and secondary oxidation products in *Polypterus bichir bichir* fish oil.

**TABLE 3:** Effect of reheating on cow meat oil quality.

Samples	Peroxide value (meq O_2_/kg)	*p*‐Anisidine value	TOTOX value
RM (Control)	0.36 ± 0.00^a^	3.00 ± 0.02^a^	3.72 ± 0.03^a^
BM (Control)	6.86 ± 1.53d^c^	10.97 ± 0.00^b^	24.70 ± 3.06^cd^
MRB1	7.16 ± 1.99^d^	15.58 ± 0.01^d^	29.91 ± 3.99^de^
MRB2	5.65 ± 0.64^c^	21.19 ± 2.02^f^	32.49 ± 3.31^e^
MRB3	7.90 ± 2.35^de^	21.56 ± 2.22^f^	37.37 ± 6.93^e^
MRF1	5.48 ± 0.50^c^	12.64 ± 0.57^c^	23.62 ± 1.58^c^
MRF2	5.156 ± 0.63^c^	12.90 ± 0.21^c^	23.22 ± 1.47^c^
MRF3	5.96 ± 0.55^c^	15.55 ± 1.32^d^	27.48 ± 2.42^d^
MROR1	7.92 ± 1.12^de^	13.07 ± 0.65^c^	28.93 ± 2.89^de^
MROR2	13.35 ± 4.37^f^	20.23 ± 1.74^f^	46.95 ± 5.66^f^
MROR3	2.64 ± 1.05^b^	24.02 ± 1.56^f^	29.31 ± 3.61^d^
MRMW1	8.88 ± 0.66^e^	13.60 ± 0.81^c^	31.45 ± 2.13^e^
MRMW2	6.20 ± 0.17^dc^	17.52 ± 0.41^e^	19.94 ± 0.76^b^
MRMW3	5.33 ± 0.27^c^	22.74 ± 1.47^f^	33.41 ± 2.01^e^

*Note*: Data are presented as mean ± SD (*n* = 2) (a‐f) Values within the same column with different superscripts are significantly (*p* < .05) different.

Abbreviations: BM, boiled control (boiled meat); MFR1, meat reheated by frying on day 1; MFR2, meat reheated by frying on day 2; MFR3, meat reheated by frying on day 3; MRB1, meat reheated by boiling on day 1; MRB2, meat reheated by boiling on day 2; MRB3, meat reheated by boiling on day 3; MRMW1, meat reheated by microwaving on day 1; MRMW2, meat reheated by microwaving on day 2; MRMW3, meat reheated by microwaving on day 3; MROR1, meat reheated by oven roasting on day 1; MROR2, meat reheated by oven roasting on day 2; MROR3, meat reheated by oven roasting on day 3; RM, boiled control (raw meat).

### 3.5 Functional properties

#### 3.5.1 Water‐ and oil‐holding capacities

**FIGURE 2:**
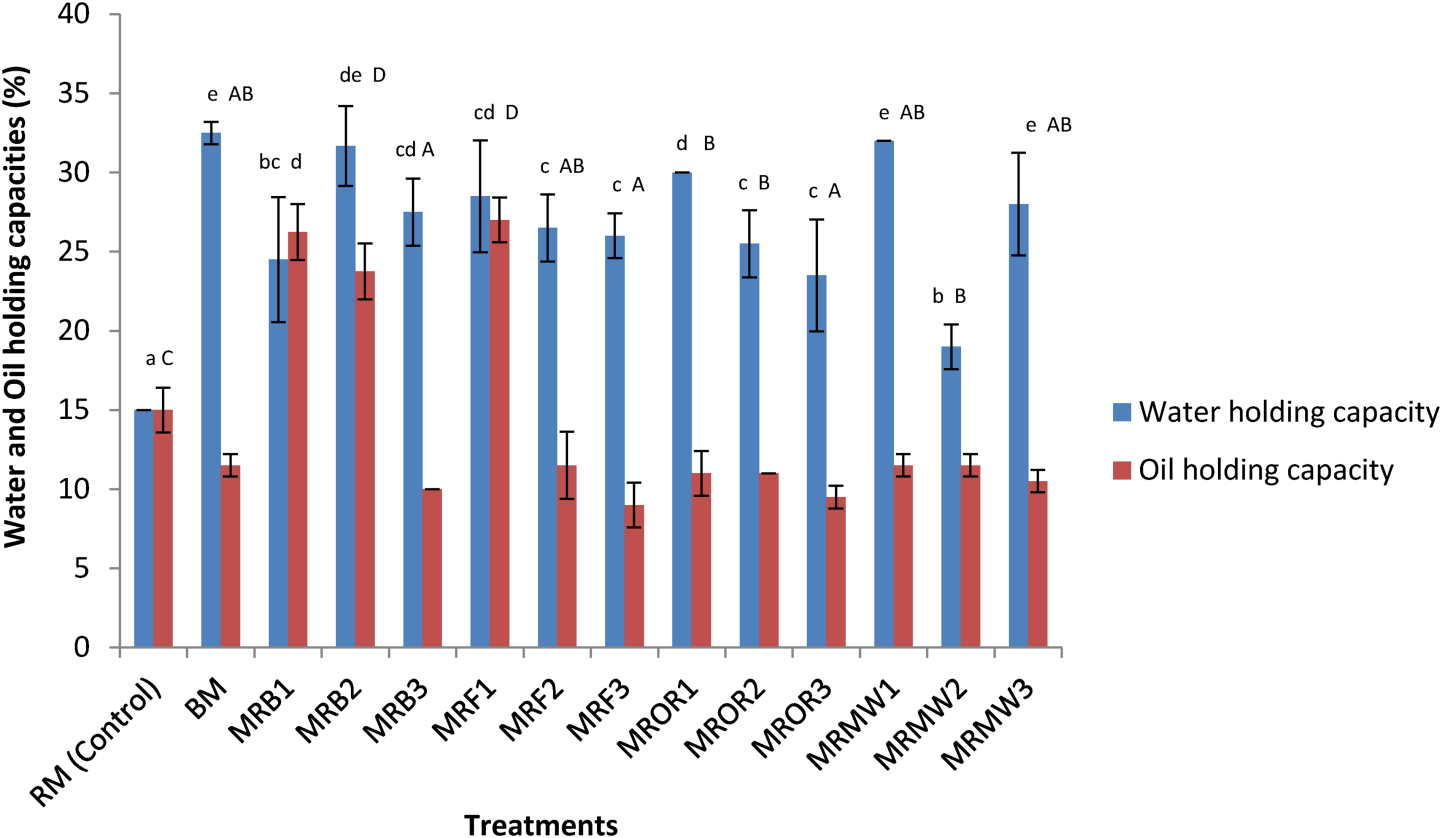
Effect of reheating on the water‐ and oil‐holding capacity of meat proteins. Data are presented as mean ± SD (*n* = 2) (a‐e; A‐D) Values for the same parameter with different superscripts are significantly (*p* < .05) different. BM, boiled control (boiled meat); MFR1, meat reheated by frying on day 1; MFR2, meat reheated by frying on day 2; MFR3, meat reheated by frying on day 3; MRB1, meat reheated by boiling on day 1; MRB2, meat reheated by boiling on day 2; MRB3, meat reheated by boiling on day 3; MRMW1, meat reheated by microwaving on day 1; MRMW2, meat reheated by microwaving on day 2; MRMW3, meat reheated by microwaving on day 3; MROR1, meat reheated by oven roasting on day 1; MROR2, meat reheated by oven roasting on day 2; MROR3, meat reheated by oven roasting on day 3; RM, boiled control (raw meat).

The influence of reheating on the water‐ and oil‐holding capacities of meat powder is presented in Figure 2. A significant (*p* < .05) increase in water‐holding capacity was observed in all the processed samples compared to the raw control (RM). Their values ranged between 15% and 33% (for the WHC) which were higher than 8.67%–13.34% reported by Kandeepan et al. (2013) with meat from different groups of buffalos. These show that the processing method applied increased the ability of the meat to hold its own water and to bind to water added separately. The amount of water retained depends on the polar and nonpolar charges carried by the proteins. The high WHC obtained with processed samples suggests that they can be used in the formulation of food with high viscosity (Smith & Bowers, 1972). For the OHC, samples reheated by boiling on days 1 and 2 (MRB1 and MRB2) and those reheated by frying on day 1 (MRF1) presented significantly (*p* < .05) higher OHC compared to the control and other processed samples. The other processed samples presented significantly (*p* < .05) lower OHC compared to the control. The oil‐holding capacity informs on the ability of flour or powder to enhance the organoleptic properties of foods through flavor retention (Adebowale & Lawal, 2004). Meat powders obtained from sample reheated by boiling on days 1 and 2 (MRB1 and MRB2) and frying on day 1 (MRF1) were found to significantly (*p* < .05) increase the OHC of cow meat powder, while the other treatments significantly (*p* < .05) decreased this parameter compared to the controls. The significant (*p* < .05) decrease in this parameter observed with some reheating methods can be explained by the distortion of protein structure facilitating them to aggregate. Similar observations were made by Djikeng et al. (2022) with processed snail meat samples. The fact that some treatments significantly (*p* < .05) increased the OHC of cow meat powder is in agreement with the findings of Chau and Cheung (1998) who reported that high processing temperature facilitates the hydrophobicity by presenting apolar groups. This characteristic makes reheating by boiling on day 1 plus reheating by frying useful in food technology whenever oil needs to be retained. The OHC obtained in this study varies from 8% to 26%, which was not far from 10%–25% reported by Djikeng et al. (2022) with snail meat powder.

#### 3.5.2 Loose and packed bulk densities

**FIGURE 3:**
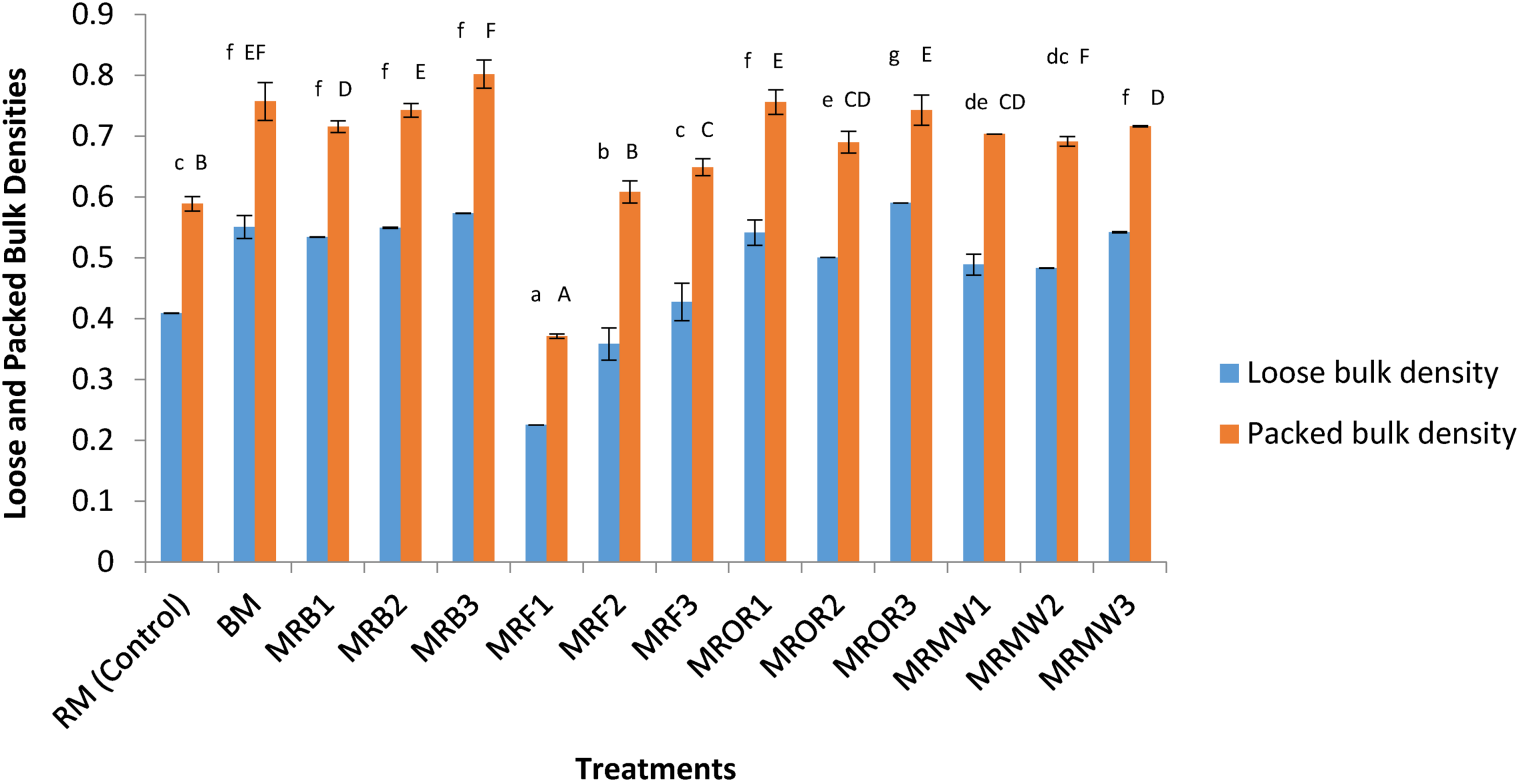
Effect of reheating on the bulk and loose densities of meat proteins. Data are presented as mean ± SD (*n* = 2) (a‐f; A‐F) Values for the same parameter with different superscripts are significantly (p < .05) different. BM, boiled control (boiled meat); MFR1, meat reheated by frying on day 1; MFR2, meat reheated by frying on day 2; MFR3, meat reheated by frying on day 3; MRB1, meat reheated by boiling on day 1; MRB2, meat reheated by boiling on day 2; MRB3, meat reheated by boiling on day 3; MRMW1, meat reheated by microwaving on day 1; MRMW2, meat reheated by microwaving on day 2; MRMW3, meat reheated by microwaving on day 3; MROR1, meat reheated by oven roasting on day 1; MROR2, meat reheated by oven roasting on day 2; MROR3, meat reheated by oven roasting on day 3; RM, boiled control (raw meat).

Figure 3 presents the effect of different reheating conditions on the loose and packed bulk densities of cow meat powders. The determination of the density is a useful parameter in food packaging. Results generally showed that reheating significantly (*p* < .05) increases the packed and loose bulk densities of cow meat powder. This is interesting in the sense that cow meat powder can be packaged and transported. From a nutritional standpoint, the increase in loose bulk density reflects good digestibility of food and increases nutrient and energy value, which are important in food formulation (Osundahunsi & Aworh, 2002). Loose and packed bulk densities rely a lot on particle size. When particle size is low, loose and packed bulk densities significantly (*p* < .05) increase as observed in this study. This can be explained by the increase in contact surface, cohesive, and frictional forces that facilitate resisting flowability and compaction ability and shape of particles (Perea‐Flores et al., 2021). The loose and packed bulk densities obtained in this study were ranged between 0.2 and 0.8, which were in agreement with findings of Djikeng et al. (2022) with snail meat powder.

#### 3.5.3 Porosity and Hausner ratio

**FIGURE 4:**
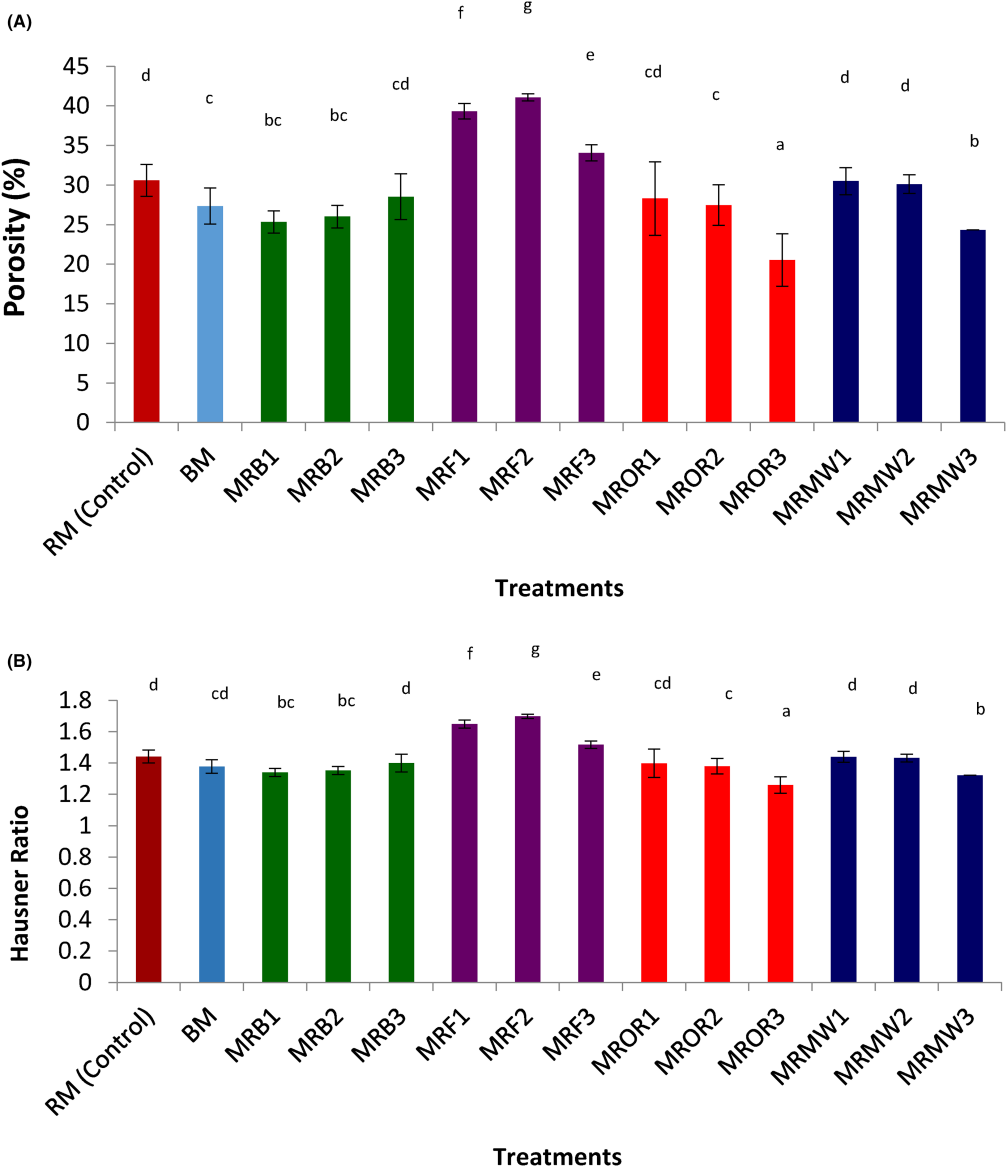
(A, B) Effect of reheating on the porosity (A) and Hausner ratios (B) of cow meat powder. Data are presented as mean ± SD (*n* = 2) (a‐g) Values for the same parameter with different superscripts are significantly (*p* < .05) different. BM, boiled control (boiled meat); MFR1, meat reheated by frying on day 1; MFR2, meat reheated by frying on day 2; MFR3, meat reheated by frying on day 3; MRB1, meat reheated by boiling on day 1; MRB2, meat reheated by boiling on day 2; MRB3, meat reheated by boiling on day 3; MRMW1, meat reheated by microwaving on day 1; MRMW2, meat reheated by microwaving on day 2; MRMW3, meat reheated by microwaving on day 3; MROR1, meat reheated by oven roasting on day 1; MROR2, meat reheated by oven roasting on day 2; MROR3, meat reheated by oven roasting on day 3; RM, boiled control (raw meat).

Porosity and Hausner ratio were calculated from the loose and packed bulk densities. Porosity influences the storage, transportation, and packaging conditions of food (Drakos et al., 2019), while the Hausner ratio measures the compaction and compression of particles friction (Shumaila et al., 2015). The effect of reheating on the Hausner ratio and porosity of cow meat powder are presented in Figure 4A,B. The porosity was found to considerably (*p* < .05) increase with frying treatments, while it decreased with other treatments compared to the controls (BM and RM). Similar observations were made with Hausner ratio. The porosity and Hausner ratio were found to range between 20% and 40% and 1.3 and 1.8, which were, respectively, higher than 5%–30% and 1.1–1.3 obtained by Djikeng et al. (2022) with snail meat powder. Frying was found to significantly (*p* < .05) increase these parameters in cow meat compared to other reheating methods. Similar results were obtained by Djikeng et al. (2022) in fried snail meat samples. The Hausner ratio obtained in this study was greater than 1.25, indicating a fair to free‐flow powder. From these results, it can be observed that frying makes cow meat powder to be packaged easily, stored, and transported, which can be useful in food industries for food formulation.

#### 3.5.4 Swelling capacity

**FIGURE 5:**
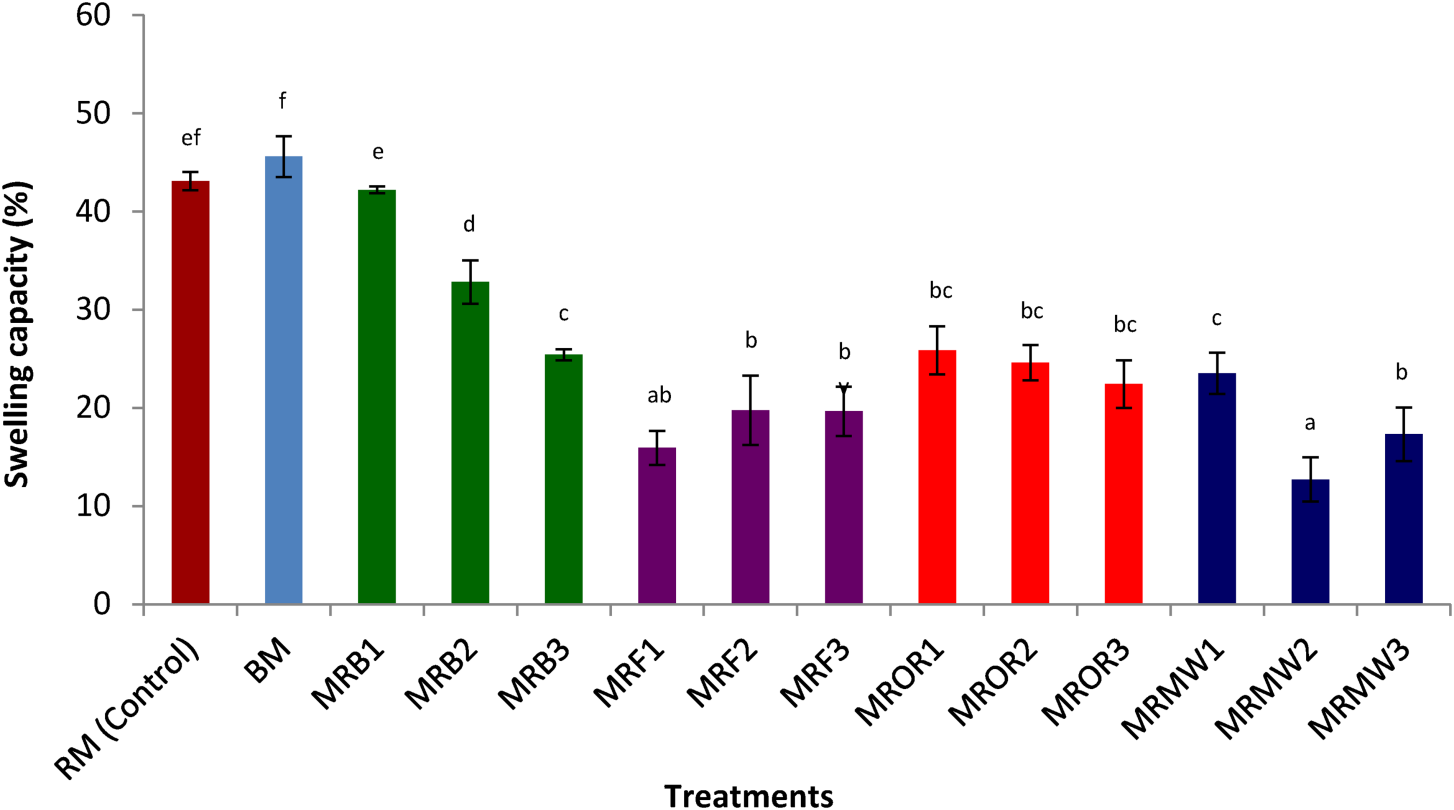
Effect of reheating on the swelling capacity of cow meat proteins. Data are presented as mean ± SD (*n* = 2) (a‐f) Values with different superscripts are significantly (*p* < .05) different. BM, boiled control (boiled meat); MFR1, meat reheated by frying on day 1; MFR2, meat reheated by frying on day 2; MFR3, meat reheated by frying on day 3; MRB1, meat reheated by boiling on day 1; MRB2, meat reheated by boiling on day 2; MRB3, meat reheated by boiling on day 3; MRMW1, meat reheated by microwaving on day 1; MRMW2, meat reheated by microwaving on day 2; MRMW3, meat reheated by microwaving on day 3; MROR1, meat reheated by oven roasting on day 1; MROR2, meat reheated by oven roasting on day 2; MROR3, meat reheated by oven roasting on day 3; RM, boiled control (raw meat).

Swelling capacity is an important parameter considered in food formulation, especially in the baking and food processing industry. It characterizes the presence of low‐energy bonds between substances in food samples. Figure 5 presents the effect of reheating on the swelling capacity of cow meat powder. All the treatments applied significantly (*p* < .05) decreased the swelling capacity of cow meat powder. However, this decrease was lower in boiled samples. Reheating was found to significantly (*p* < .05) reduce this parameter compared to the control. Only samples boiled for maximum 2 days exhibited higher swelling capacity compared to the other reheating methods. These results are not in agreement with the findings of Djikeng et al. (2022) who reported that thermal processing significantly (*p* < .05) increases the swelling capacity of snail meat powder. Generally, it can be concluded that constant reheating of cow meat damages this property. It has been reported that swelling capacity of proteins can be influenced by temperature, particle size, ionic strength, and the protein source (Pomeranz, 1985).

#### 3.5.5 pH and titratable acidity (TTA)

**FIGURE 6:**
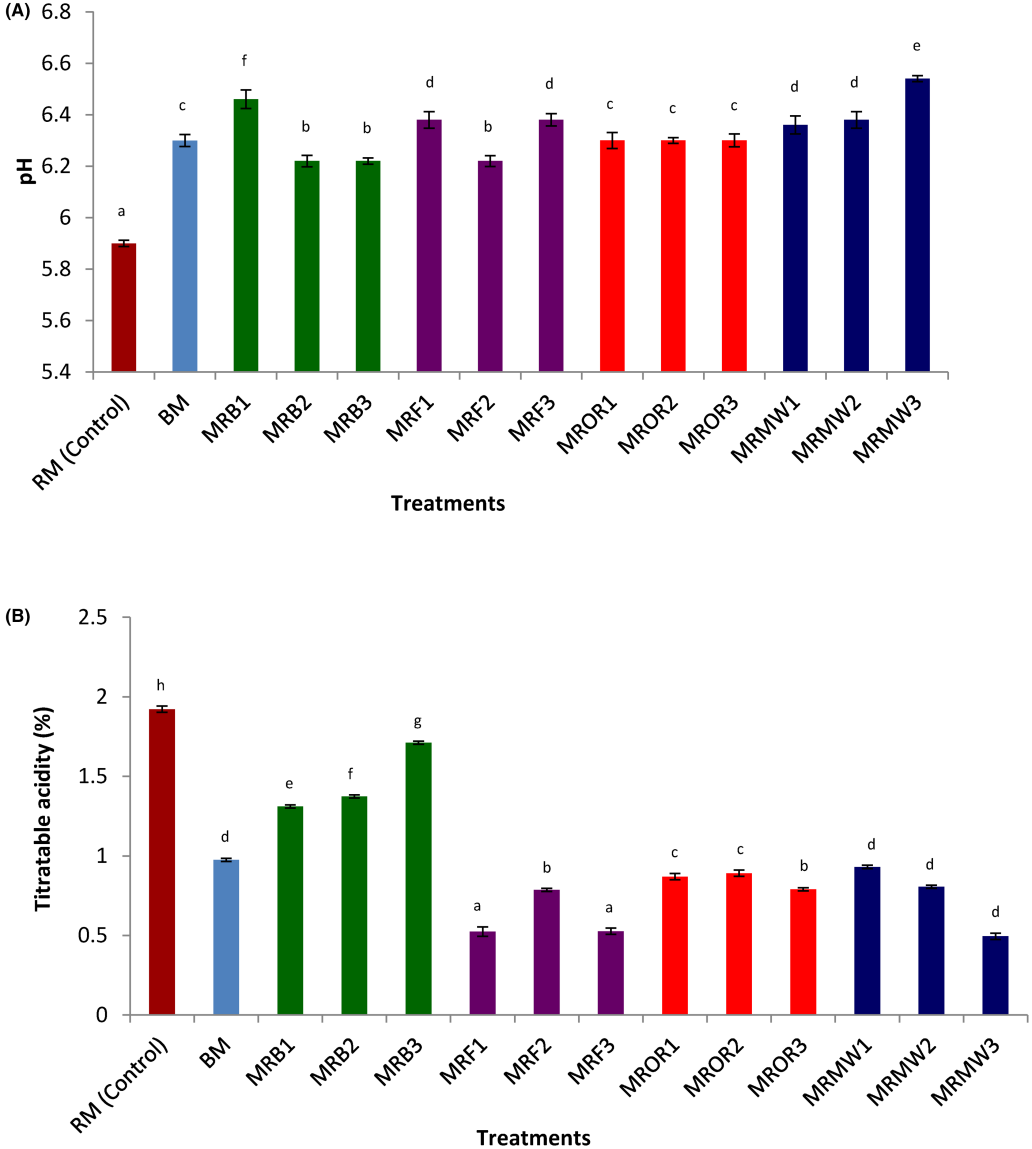
(A, B) Effect of reheating on the pH (A) and titratable acidity (B) of cow meat powder. Data are presented as mean ± SD (*n* = 2) (a‐g) Values for the same parameter with different superscripts are significantly (*p* < .05) different. BM, boiled control (boiled meat); MFR1, meat reheated by frying on day 1; MFR2, meat reheated by frying on day 2; MFR3, meat reheated by frying on day 3; MRB1, meat reheated by boiling on day 1; MRB2, meat reheated by boiling on day 2; MRB3, meat reheated by boiling on day 3; MRMW1, meat reheated by microwaving on day 1; MRMW2, meat reheated by microwaving on day 2; MRMW3, meat reheated by microwaving on day 3; MROR1, meat reheated by oven roasting on day 1; MROR2, meat reheated by oven roasting on day 2; MROR3, meat reheated by oven roasting on day 3; RM, boiled control (raw meat).

The impact of reheating on the pH and titratable acidity of cow meat powder is presented in Figure 6A,B. All the treatments applied have significantly (*p* < .05) increased the pH of the meat. Generally, processing significantly (*p* < .05) decreased the titratable acidity of cow meat compared to the raw control (RM). Fried samples (MFR) exhibited the lowest titratable acidity among the processed samples. Samples reheated by boiling presented the highest TTA. The pH and titratable acidity of the powder obtained from reheated cow meat were found to range between 5.9% and 6.4% and 0.5% and 2%, respectively. The pH range obtained in this study was close to 5.59–5.59 reported by Kandeepan et al. (2013) with cow meat. It is well known that lower pH values and high acidity are essential for the inhibition of the development of microorganisms in food products as well as the inhibition of enzymatic reactions. All the reheating methods applied were found to significantly (*p* < .05) increase the pH values and reduce the titratable acid of the powder obtained from cow meat. This suggests that alteration methods by microorganisms can easily take place in these samples after they have been reheated. These results are in line with the findings of Djikeng et al. (2022) which observed similar trend in snail meat powder after they were thermally processed.

### 3.6 Bacteria count

The impact of different reheating methods on the bacterial load of cow meat is exhibited in Table 4. The boiled control (BM), the meat reheated by boiling on days 1 and 2 (MRB), and the ones reheated by oven roasting on day 1 (MROR) presented significantly (*p* < .05) higher bacterial count compared to the raw control (RM). The bacterial population was, however, reduced with reheating cycles. The decrease in microbial population with processing recorded with each reheating process can be the result of the temperature applied which had a significant effect on the destruction of contaminating bacteria. Generally, the results obtained showed that the reheated meat samples were still acceptable at the bacterial load standpoint, as their bacterial population was lower than 10^6^ CFU/g, as recommended by Egyptian Organization for Standardization (EOS) (2005). The significantly (*p* < .05) higher microbial load obtained in some samples can be due to the poor handling of meat during processing. Similar results were obtained by Ibrahim et al. (2020) when evaluating the impact of cooking on the bacteriological properties of meat. Previous reports have shown the use of methods different from reheating in meat preservation, especially raw meat. Akacha et al. (2022) reported that heteropolysaccharides extracted from *Loburia maritime* significantly improve the shelf life of beef. In the same line, Hsouna et al. (2020) demonstrated that a novel *Triticum durum* Annexin 12 protein inhibits the development of *Listeria monocytogenes* in minced beef meat. Thiamine was also demonstrated to be a biopreservative and antimicrobial agent in minced beef meat during storage compared to the control (Hsouna et al., 2022).

**TABLE 4:** Effect of reheating on the bacterial count of cow meat.

Samples	Total bacteria count (CFU/g)
RM (Control)	25 × 10^3^
BM (Control)	54 × 10^3^
MRB1	34 × 10^3^
MRB2	84 × 10^3^
MRB3	23 × 10^3^
MRF1	25 × 10^3^
MRF2	5 × 10^3^
MRF3	1 × 10^3^
MROR1	135 × 10^3^
MROR2	26 × 10^3^
MR0R3	6 × 10^3^
MRMW1	6 × 10^3^
MRMW2	1 × 10^3^
MRMW3	16 × 10^3^

Abbreviations: BM, boiled control (boiled meat); MFR1, meat reheated by frying on day 1; MFR2, meat reheated by frying on day 2; MFR3, meat reheated by frying on day 3; MRB1, meat reheated by boiling on day 1; MRB2, meat reheated by boiling on day 2; MRB3, meat reheated by boiling on day 3; MRMW1, meat reheated by microwaving on day 1; MRMW2, meat reheated by microwaving on day 2; MRMW3, meat reheated by microwaving on day 3; MROR1, meat reheated by oven roasting on day 1; MROR2, meat reheated by oven roasting on day 2; MROR3, meat reheated by oven roasting on day 3; RM, boiled control (raw meat).

## 4 CONCLUSION

The objective of this study was to evaluate the effect of different reheating methods on the proximate composition, mineral content, oil quality, and functional and microbiological properties of cow meat. Results revealed that frequent reheating by boiling/steaming is the method applied in households for food preservation. Meat reheated by boiling and microwaving reduces the protein content of cow meat while frying and oven roasting increase it. The reheating methods applied generally reduce the mineral content of cow meat. The quality of cow meat oil significantly reduces during the reheating processes and time. Reheating significantly improves the water‐holding capacity, loose and packed bulk densities, and the pH values while only reheating by frying increases the porosity and Hausner ratio of cow meat powder. All the reheating methods significantly reduce the swelling capacity and titratable acidity of the meat. Reheating generally reduces the bacteria population of cow meat even though they were still within acceptable range which is below 10^6^ CFU.

## AUTHOR CONTRIBUTIONS

**Ombotoh Sabastin Nanje:** Conceptualization (equal); data curation (equal); formal analysis (equal); methodology (equal); writing – original draft (equal); writing – review and editing (equal). **Veshe‐Teh Zemoh Sylvia Ninying:** Conceptualization (equal); data curation (equal); investigation (equal); methodology (equal); resources (equal); writing – original draft (equal); writing – review and editing (equal). **Fabrice Tonfack Djikeng:** Conceptualization (lead); data curation (equal); formal analysis (equal); investigation (equal); methodology (equal); resources (equal); supervision (equal); validation (equal); visualization (equal); writing – original draft (equal); writing – review and editing (equal). **Theresia Azia Morfor:** Conceptualization (equal); data curation (equal); formal analysis (equal); investigation (equal); methodology (equal); supervision (equal); validation (equal); visualization (equal); writing – original draft (equal). **Aduni Ufuan Achidi:** Conceptualization (equal); data curation (equal); formal analysis (equal); investigation (equal); methodology (equal); resources (equal); supervision (equal); validation (equal); visualization (equal); writing – original draft (equal); writing – review and editing (equal).

## CONFLICT OF INTEREST STATEMENT

The authors declare that they have no known competing financial interests or personal relationships that could have appeared to influence the work reported in this paper.

## ETHICS STATEMENT

This is to inform you that in this study, we have not been involved in any animal and human studies.

## Data Availability

The authors confirm that the data supporting the findings of this study are available within the article.
